# Incorporating Breast Anatomy in Computational Phenotyping of Mammographic Parenchymal Patterns for Breast Cancer Risk Estimation

**DOI:** 10.1038/s41598-018-35929-9

**Published:** 2018-11-30

**Authors:** Aimilia Gastounioti, Meng-Kang Hsieh, Eric Cohen, Lauren Pantalone, Emily F. Conant, Despina Kontos

**Affiliations:** Department of Radiology, Perelman School of Medicine, University of Pennsylvania, Philadelphia, PA 19104 USA

## Abstract

We retrospectively analyzed negative screening digital mammograms from 115 women who developed unilateral breast cancer at least one year later and 460 matched controls. Texture features were estimated in multiple breast regions defined by an anatomically-oriented polar grid, and were weighted by their position and underlying dense versus fatty tissue composition. Elastic net regression with cross-validation was performed and area under the curve (AUC) of the receiver operating characteristic (ROC) was used to evaluate ability to predict breast cancer. We also compared our anatomy-augmented features to current state-of-the-art in which parenchymal texture was assessed without considering breast anatomy and evaluated the added value of the extracted features to breast density, body-mass-index (BMI) and age as baseline predictors. Our anatomy-augmented texture features resulted in higher discriminatory capacity (AUC = 0.63 vs. AUC = 0.59) when breast anatomy was not considered (*p* = 0.021), with dense tissue regions and the central breast quadrant being more heavily weighted. Texture also improved baseline models (from AUC = 0.62 to AUC = 0.67, *p* = 0.029). Our findings suggest that incorporating breast anatomy information could augment imaging markers of breast cancer risk with the potential to improve personalized breast cancer risk assessment.

## Introduction

Breast cancer risk assessment has become increasingly important for forming tailored breast cancer screening and prevention strategies^1,2^. Full-field digital mammography (FFDM), routinely used for breast cancer screening^3^, has demonstrated substantial potential in providing quantitative image-derivable measures which relate to breast cancer risk^4–7^. Mammographic density has been the most established such measure and has been shown to be a strong independent risk factor for breast cancer^8,9^.

Going beyond breast density, recent studies also suggest that parenchymal texture, which reflects the heterogeneity of the breast parenchymal pattern, plays a complementary role in breast cancer risk assessment by capturing information that is not reflected by mammographic density alone or other established risk factors^10^. This research field, aiming to translate mammographic images to computational imaging phenotypes of breast cancer risk, is rapidly evolving and several computerized methodologies have been developed to quantify the properties of the breast parenchymal pattern using texture descriptors^10^.

Although useful in risk prediction, parenchymal texture measurements do not generally incorporate breast morphology and anatomy — *i.e*., the shape, size, and tissue structure of the specific breast analyzed. Breast anatomy may, however, be an important component in quantification of breast parenchymal patterns. For instance, texture characterization adapted to the largely variable breast morphology would allow establishing anatomical correspondences across mammograms and, therefore, generate more comparable textural measurements across subjects. In addition, and perhaps most importantly, parenchymal characteristics from different areas of the breast may contribute differently towards the risk for developing breast cancer. For example, a recent study^11^ showed significant associations between breast cancer and the relative spatial distribution of dense versus fatty regions within the breast, with fatty tissue in the lower quadrants and the absence of density in the retromammary space being protective against breast cancer. Moreover, the central breast area (CBA) and the upper-outer area (UOA) of the breast have been associated with a higher frequency by location of breast cancers^12^ and with early changes in texture due to breast cancer development^13^. These findings suggest that inherent breast tissue properties predisposing women to higher risk of breast cancer may not be uniformly expressed in the breast parenchyma and, therefore, allowing breast morphology and anatomy to drive texture measurements could augment their predictive capacity.

The purpose of our study was to evaluate whether incorporating breast anatomy information can strengthen the associations of mammographic parenchymal texture phenotypes with breast cancer risk. Towards this end, we introduce a computational breast-anatomy-driven approach for extracting parenchymal texture features and evaluate their predictive value in a case-control study with full-field digital mammograms.

## Methods

### Study population

In this IRB-approved (University of Pennsylvania; Protocol #: 825735), HIPAA-compliant study under a waiver of consent, we retrospectively analyzed a case-control sample of raw (*i.e*., ‘FOR PROCESSING’) bilateral digital mammograms. Briefly, cases (*n* = 116) included all women diagnosed with unilateral breast cancer between July 2012 and December 2014 (biopsy and state registry confirmed cancer diagnosis) who also had negative screening mammograms available at our institution at least one year prior to their diagnosis. One case was excluded due to the presence of bilateral breast implants. For all the remaining 115 cases, we retrieved the earliest negative screening study with raw mammograms available (average time from screening to diagnosis: 1.9 years ± 0.7) which was used for our analysis. Eligible controls were women who had a negative routine screening exam during the same period and confirmed negative one-year follow-up. Following case-control matching without replacement, controls were matched to cases on age at screening (within 5-year intervals), ethnicity and screening exam date (within 1 year) at a 4:1 ratio (*n* = 460), yielding a total sample of 575 women. Briefly, cases and controls were randomly ordered. Then, for each case, eligible controls were identified as controls matching to cases on the specified matching criteria and four of them were randomly selected. Since we were matching without replacement, matched controls were not available to serve as matches for subsequent cases. This process was repeated until four matches were found for each cancer case.

For the purposes of our study, we used the mediolateral-oblique (MLO) view of the available digital mammograms as it visualizes the maximum amount of breast tissue and can potentially better define anatomically the breast quadrants. All images were acquired using Selenia Dimensions (Hologic, Inc., Bedford, MA, USA) units. Breast Imaging-Reporting and Data System (BI-RADS) density classification and body-mass index (BMI) were extracted from the archived clinical records and the radiologists’ screening reports for all women. Fully-automated quantitative measurements of breast percent density (PD) were also obtained for all images in this study, using the publicly available “Laboratory for Individualized Breast Radiodensity Assessment” (LIBRA, v.1.0.4) software^14^ which provides area-based breast PD (APD) measurements, and the commercially available software package Quantra ^TM^ (Hologic Inc., Bedford, MA, v.2.2.1), which allows for APD and volumetric breast PD (VPD) assessment.

### Computational parameterization of breast anatomy

**Figure 1:**
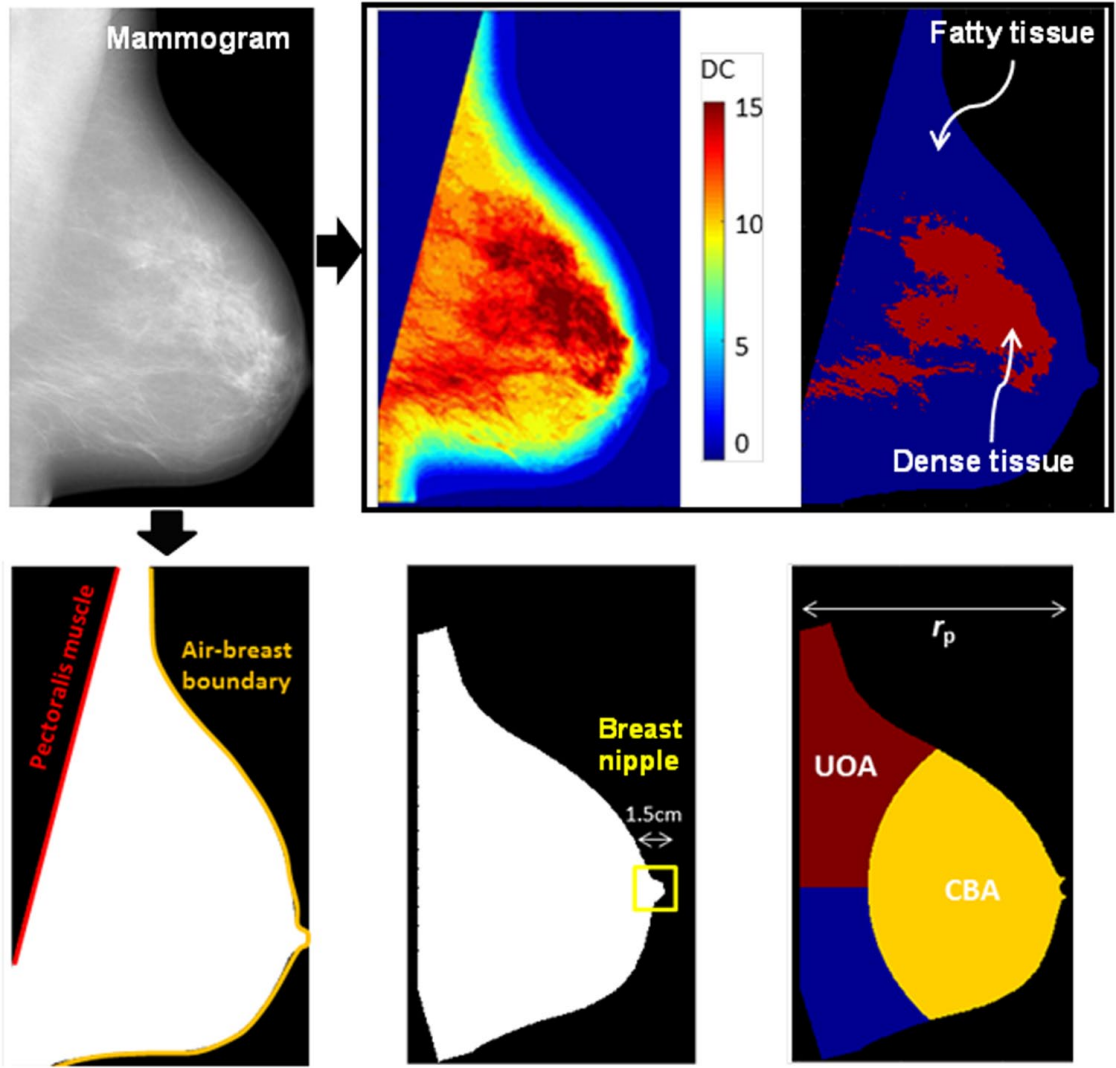
Breast anatomy and morphology captured by landmarks and key sub-regions of the breast parenchyma.

To incorporate breast anatomy in quantitative computational metrics, first several landmarks and sub-regions of the breast parenchyma were defined in each 2D FFDM image: (a) the air-breast boundary, (b) the pectoralis muscle, (c) regions of predominantly dense versus predominantly fatty tissue, (d) the nipple, and (e) the UOA and CBA (Fig. 1).

The anatomical components (a)–(c) were identified using LIBRA^14,15^. Specifically, LIBRA delineates the air-breast boundary using automated gray-level thresholding, and extracts the pectoralis muscle region by applying a straight-line Hough transform^16^. To identify dense versus fatty tissue areas, LIBRA uses adaptive fuzzy c-means clustering to partition the breast into density clusters (DCs) of similar gray-level intensity, which are then aggregated into the final dense tissue segmentation.

For landmarks (d)–(e), the breast image was first rotated, and then flipped for right breasts, so that the pectoralis muscle was vertically aligned on the left side of all images. Then, the nipple was approximated as the rightmost circular region of the breast bounded by a rectangle of maximum size 1.5 cm in both dimensions. Lastly, two breast sub-regions were identified: for *r*_P_ equal to the perpendicular distance from the pectoralis muscle to the nipple, the CBA was defined as that part of the breast at a radial distance less than *f* *r_P_ from the nipple, and the UOA as that part of the breast lying above the nipple and further than *f* *r_P_ from the nipple. The parameter *f*, was implemented to range within [0.5, 1] so that CBA adequately covers the central part of the breast, defining the relative sizes of the CBA and the UOA.

**Figure 2:**
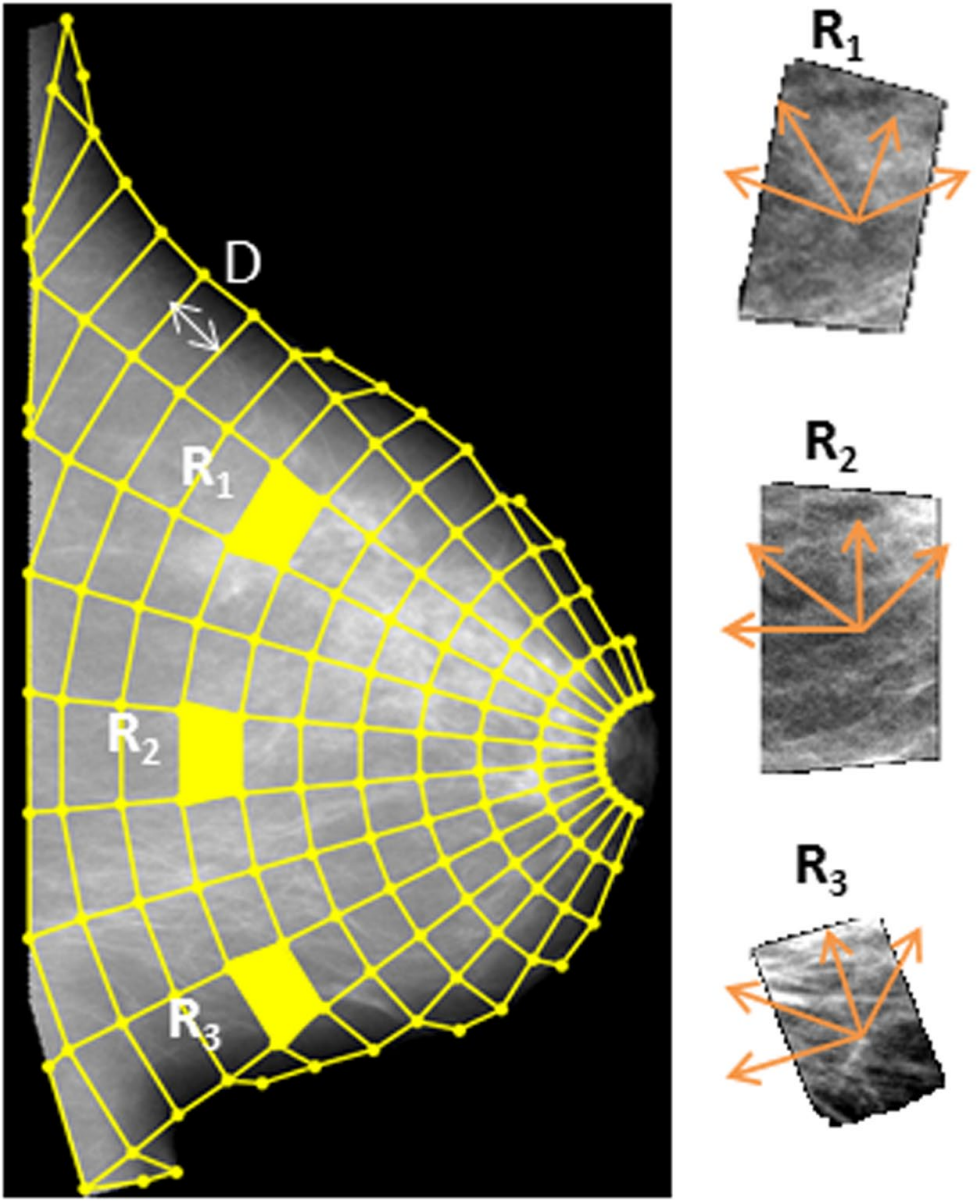
Anatomical sampling of the breast. Polar grid fitted to the morphology of the particular breast and morphology-aligned orientations for texture feature calculations.

A polar grid of a radius unit equal to *D*, centered on the nipple, was then overlaid on the mammographic image (Fig. 2). The polar grid was fitted to the shape and size of the individual breast as well as to an approximation of the ductal distribution extending from the nipple posteriorly and perpendicular to the pectoralis muscle in a radial fashion. Centering on the nipple allowed for denser sampling in the retro-areolar region of the breast, which typically contains more complex parenchymal tissue patterns than the breast areas closer to the pectoralis muscle^17^.

### Anatomically-oriented texture feature extraction

Within each region defined by the polar grid, we estimated a total of 34 established texture descriptors (Table 1 and *Supplementary Note N1* for detailed mathematical formulations), including gray-level histogram, co-occurrence, run-length, and structural features, all of which have been previously used for mammographic pattern analysis and breast cancer risk assessment^10^. Briefly, gray-level histogram features are common first-order statistics which describe the distribution of gray-level intensity. The co-occurrence features also consider the spatial relationships of pixel intensities in specified directions and are based on the gray-level co-occurrence matrix (GLCM) which encodes the relative frequency of neighboring intensity values. Run-length features capture the coarseness of texture in specified directions by measuring strings of consecutive pixels which have the same gray-level intensity along specific linear orientations. Using the polar grid, the axes for calculating the co-occurrence and run-length features were aligned with the structure of the parenchymal tissue and ductal structures (Fig. 2). Finally, structural features capture the architectural composition of the parenchyma by characterizing the tissue complexity, the directionality of flow-like structures in the breast, and intensity variations between central and neighboring pixels. A total of 34 feature maps were computed (i.e., one for each feature), which captured the spatial distribution of the corresponding parenchymal texture measurements as sampled by the polar grid over the entire breast.

**Table 1:** Parenchymal texture features (TF) measured in each anatomically-defined region.

*Gray-level Histogram*	
TF1	5^th^ Percentile
TF2	5^th^ Mean
TF3	95^th^ Percentile
TF4	95^th^ Mean
TF5	Entropy
TF6	Kurtosis
TF7	Max
TF8	Mean
TF9	Min
TF10	Sigma
TF11	Skewness
TF12	Sum
TF13	Median
***Co-occurrence***	
TF14	Contrast
TF15	Correlation
TF16	Homogeneity
TF17	Energy
TF18	Entropy
TF19	Inverse Difference Moment
TF20	Cluster Shade
***Run-length***	
TF21	Short Run Emphasis
TF22	Long Run Emphasis
TF23	Gray Level Non-uniformity
TF24	Run Length Non-uniformity
TF25	Run Percentage
TF26	Low Gray Level Run Emphasis
TF27	High Gray Level Run Emphasis
TF28	Short Run Low Gray Level Emphasis
TF29	Short Run High Gray Level Emphasis
TF30	Long Run Low Gray Level Emphasis
TF31	Long Run High Gray Level Emphasis
***Structural***	
TF32	Edge-enhancing index
TF33	Box-Counting Fractal Dimension
TF34	Local Binary Pattern

### Texture feature summarization weighted by breast anatomy

**Figure 3:**
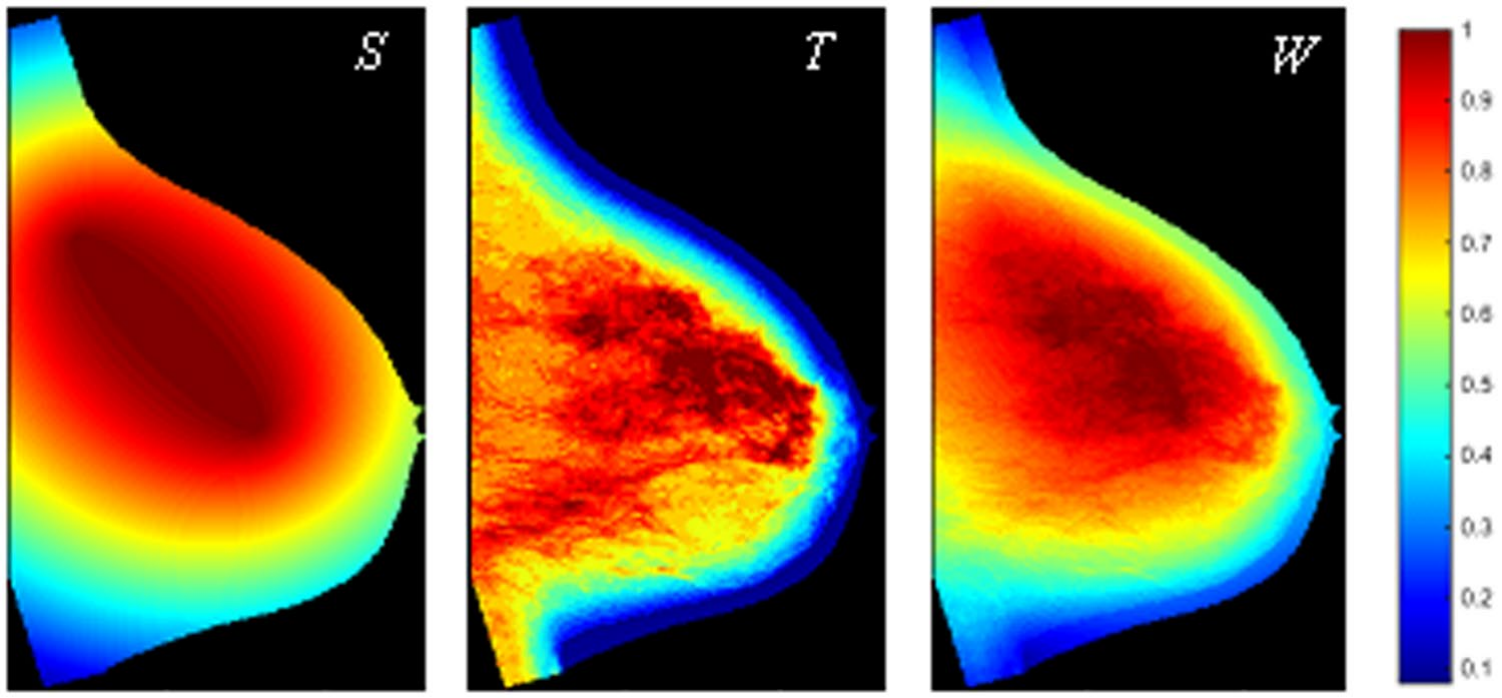
Generating the weight of each region. Example of weight map (*W*, for *c* = 0.8) representing the anatomical structure (*S*, $a$ = 0.5) and the underlying tissue composition (*T*, *b* = 1) of the breast.

As the final step, we generated a weight map which assigned a weight to each region; this weight map was, then, region-wise multiplied to the 34 original texture feature maps to generate a set of 34 weighted texture feature maps. Our design of the weight map was motivated by studies associating the CBA and UOA with potential specific roles in breast cancer development^12,13^, and by work investigating biologic correlates of tissue composition with breast cancer development^11,18–20^. Therefore, the weight map (*W*) was designed as a combination of weights due to the region’s anatomical position (*S*), and weights due to the underlying tissue composition (*T*) of each region (Fig. 3). The *S* component, representing the coding of the anatomical location, was based on the relative distance from the CBA and UOA centroids and assigned larger weights to regions within those anatomical quadrants, with parameter $a$ tuning the role of CBA versus UOA. The component *T*, representing the coding of the tissue composition, reflected the density clusters generated by LIBRA (Fig. 1), with parameter *b* indicating whether a higher weight was assigned to areas of dense tissue or fatty tissue. The relative importance of weights *S* and *T* when merged to the final weight map was defined by parameter *c*. (See *Supplementary Note N2* for detailed definitions.)

For each weighted texture feature map we estimated the mean value of the corresponding weighted feature across all regions (labeled “TF#_mean”), and the standard deviation (labeled “TF#_std”). These constituted the breast’s texture signature: a 68-element feature vector corresponding to the average and the variation of each of the weighted texture feature maps.

### Statistical Analysis

The optimization of our anatomy-driven approach for parenchymal texture analysis and breast cancer prediction involved two inter-related tasks: automatically configuring the tunable texture analysis parameters (*D, f, a, b*, and *c*) and determining the most discriminatory subset of covariates out of the 68 available texture features. To this end, for different parameter combinations (Supplementary Fig. S1), the following steps were applied. First, per-woman texture estimates were generated by averaging the corresponding bilateral breast texture signatures for each woman and were, then, z-score normalized. To remove features with little or no variability while also maintaining all different aspects of texture captured by the different features, we, then, identified pairs of features with absolute Pearson correlation greater than 0.90 and for each pair we removed the feature with the lowest variability in terms of its interquartile range (IQR)^7^. Starting from the remaining features, elastic net regression^21^ with nested cross-validation^22^ was used to build a parsimonious logistic regression model with the most discriminatory subset of covariates, where model performance was evaluated using the area under the curve (AUC) of the receiver operating characteristic (ROC) (see *Supplementary Note N3* for feature optimization details).

The model corresponding to maximum cross-validated AUC (i.e., optimized model) was compared with a model built using the same statistical approach as described above but where textural features were generated using a current state-of-the-art algorithm based on a simpler lattice approach to sample the breast without incorporating breast anatomy^23^. Briefly, in this lattice-based strategy, a regular lattice is overlaid on the mammographic image, and textural features are computed on local square regions centered on each lattice point within the breast; further, all regions are equally contributing to the breast’s texture signature which consists of the mean and the standard deviation of each feature distribution. The AUCs were compared using DeLong’s test^24^ and by estimating the model net reclassification improvement (NRI)^25^.

Finally, we assessed the potential of augmenting established risk factors by evaluating four baseline models including breast density (BI-RADS density categories, LIBRA APD, Quantra APD, or Quantra VPD) adjusted by BMI and age at screening, and tested the added value of incorporating our breast-anatomy-driven texture features. Baseline and augmented logistic regression models were fitted to obtain estimates of AUC, odds ratios (ORs), and statistical significance of predictor variables. For comparison, conventional lattice-based texture measurements^23^ were also evaluated in terms of their ability to augment established risk factors in breast cancer risk prediction.

All tests of statistical significance were at the standard *p* = 0.05 level. The breast-anatomy-driven approach was developed in Matlab R2014b (Mathworks, Natick, Mass) and statistical analysis was performed in Stata 13 (StataCorp LP, College Station, TX, USA).

## Results

There was a significant difference in Quantra VPD and a marginally significant difference in age between cases and controls in our sample (Table 2).

**Table 2:** Study sample characteristics by case-control status.

	Cases (*n* = 115)	Controls (*n* = 460)	p-value*
Breast Cancer Type			
*Invasive*	86 (75%)		
*In-situ*	29 (25%)		
Age (Mean ± SD)	59.02 y ± 11.7	56.7 y ± 11.5	0.049
BMI (Mean ± SD)	29.7 kg/m^2^ ± 6.9	29.5 kg/m^2^ ± 7.6	0.799
*missing*	0 (0%)	9 (2%)	
Ethnicity			1.000
*Caucasian*	54 (47%)	216 (47%)	
*African-American*	61 (53%)	244 (53%)	
BI-RADS Density			0.075
*Type A*	9 (7.8%)	54 (11.9%)	
*Type B*	61 (53.0%)	279 (60.7%)	
*Type C*	38 (33.0%)	123 (26.7%)	
*Type D*	3 (2.6%)	3 (0.7%)	
*missing*	4 (3.5%)	1 (0.2%)	
LIBRA Breast APD (Mean ± SD)	14.65% ± 11.83	13.81% ± 9.46	0.421
Quantra Breast APD (Mean ± SD)	17.67% ± 16.50	14.68% ± 15.48	0.068
Quantra Breast VPD (Mean ± SD)	13.57% ± 6.48	11.99% ± 6.39	0.018

SD: standard deviation; BMI: body mass index; BI-RADS: Breast Imaging Reporting and Data System; APD: Area-based breast percent density; VPD: Volumetric breast percent density.

*p-values from two-sample t-tests for continuous covariates and from Pearson chi-squared tests for ethnicity and BI-RADS density.

The best model performance for the anatomy-driven features was AUC = 0.63 (95% CI [0.59 0.69]). This was achieved for a spatially dense polar grid (*D* = 6.3 mm) and for a weight map in which dense tissue regions were assigned higher weights than fatty regions (*b* = 1 in weights *T*), and regions in the CBA were weighted more heavily than in the UOA (*a* = 0.8 in weights *S*). Each region’s location (weights *S*) was slightly less important than the region’s underlying tissue composition (weights *T*) in calculating the final weight (*c* = 0.4 in weights *W*). After excluding 17 features with low IQR, elastic net regression selected 30 of the 51 textural features for inclusion in this optimal model, including nine gray-level histogram, nine co-occurrence, eight run-length, and four structural features; among these, 14 represented mean values and 16 the variation (i.e., standard deviation) in texture feature maps (Supplementary Table S1).

**Figure 4:**
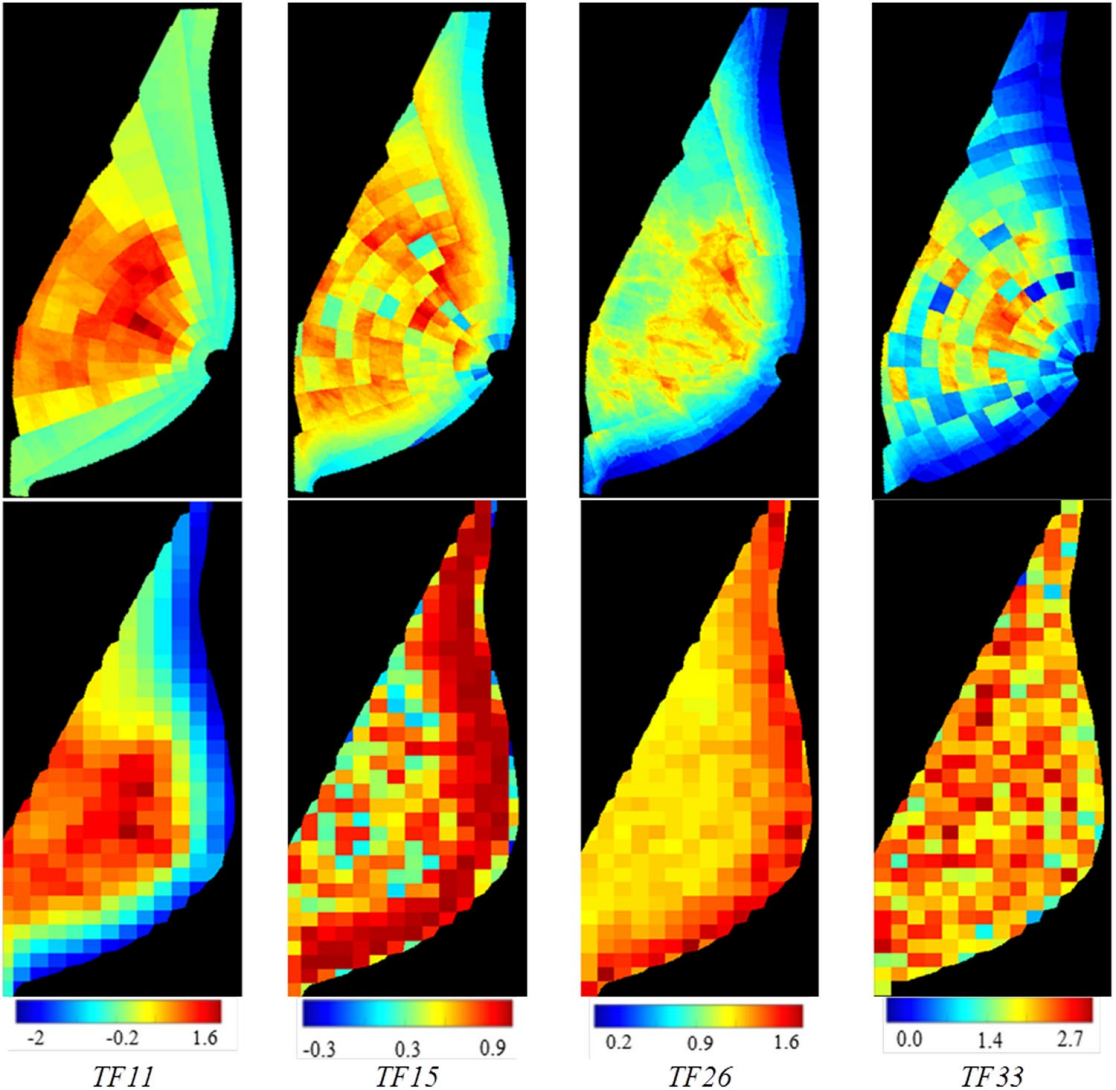
Texture feature maps for four texture descriptors. Top row: weighted values on polar grid using the proposed breast-anatomy-driven approach with the optimal set of parameters. Bottom row: non-weighted values on a regular lattice^23^.

Our anatomy-driven features outperformed the lattice-based strategy which gave AUC = 0.59 (95% CI [0.55 0.64]) (*p* = 0.021 by DeLong’s test) (Fig. 4). Based on the NRI, 20% of cases were correctly reclassified upwards and 5% of controls were correctly reclassified downwards.

Our anatomy-driven features were also able to significantly augment all four baseline models fitted with breast density, BMI and age. Specifically, when the breast-anatomy-driven texture features were added to the model based on Quantra VPD and BMI, which was the best performing baseline model (Table 3), the discriminatory capacity was significantly improved from AUC = 0.62 (95% CI [0.57 0.68]) to AUC = 0.67 (95% CI [0.60 0.72]) (*p* = 0.029 by DeLong’s test) (Table 4 and Supplementary Table S1). The lattice-based texture descriptors were also able to add significant value to the baseline models; however, they were consistently outperformed by the breast-anatomy-driven texture features (Table 4).

**Table 3:** Associations with breast cancer risk and case-control discriminatory capacity for four baseline models.

	OR	*p*-value	95% CI		AUC
**Baseline 1**					
BI-RADS Density					
*Type A*	Ref				
*Type B*	1.72	0.175	[0.79	3.78]	0.58
*Type C*	3.07	0.013	[1.26	7.47]	95% CI [0.54 0.66]
*Type D*	14.3	0.005	[2.23	91.92]	*p*^*b*^ = 0.031
BMI	1.03	0.081	[1.00	1.06]	
Age	1.03	0.004	[1.00	1.05]	
**Baseline 2**					
LIBRA APD	1.02	0.065	[1.00	1.05]	0.56
BMI	1.02	0.202	[0.99	1.06]	95% CI [0.52 0.64]
Age	1.02	0.015	[1.00	1.04]	*p*^*b*^ = 0.063
**Baseline 3**					
Quantra APD	1.02	0.006	[1.00	1.03]	0.61
BMI	1.02	0.135	[0.99	1.05]	95% CI [0.55 0.66]
Age	1.02	0.010	[1.01	1.04]	*p*^*b*^ = 0.010
**Baseline 4**					
Quantra VPD	1.06	0.001	[1.03	1.10]	0.62
BMI	1.02	0.106	[0.99	1.06]	95% CI [0.57 0.68]
Age	1.03	0.004	[1.01	1.05]	*p*^*b*^ = 0.002

Odds ratios (ORs) per standard deviation increase in the standard risk factors of breast density (BI-RADS density categories, LIBRA APD, Quantra APD, or Quantra VPD), body-mass-index (BMI) and age. Also shown: *p*-values, 95% confidence intervals (CIs), cross-validated discriminatory capacity (AUC).

*p*^*b*^: Statistical significance of baseline model.

**Table 4:** Case-control discriminatory performance of standard breast cancer risk factors combined with parenchymal texture features.

	*Breast-anatomy-driven approach*		*Lattice-based approach* ^23^		*p* ^*c*^
	AUC, 95% CI	*p* ^*a*^	AUC, 95% CI	*p* ^*a*^	
*Baseline 1* + *Texture*	0.62, 95% CI [0.56 0.67]	0.011	0.61, 95% CI [0.56 0.64]	0.029	0.051
*Baseline 2* + *Texture*	0.65, 95% CI [0.58 0.69]	0.031	0.60, 95% CI [0.56 0.65]	0.042	0.033
*Baseline 3* + *Texture*	0.66, 95% CI [0.60 0.71]	0.030	0.64, 95% CI [0.60 0.66]	0.037	0.039
*Baseline 4* + *Texture*	0.67, 95% CI [0.60 0.72]	0.029	0.64, 95% CI [0.61 0.66]	0.038	0.027

Cross-validated area under the curve (AUC) and 95% confidence intervals (CIs) for baseline models augmented by breast-anatomy-driven texture features or conventional lattice-based texture descriptors.

*p*^*a*^: *p*-value for difference in AUC from the corresponding baseline model; *p*^*c*^: *p*-value for difference in AUC between breast-anatomy-driven and lattice-based texture analysis, by DeLong’s tests.

## Discussion

Our findings suggest that incorporating breast anatomy information in mammographic phenotypes of parenchymal pattern could augment imaging markers of breast cancer risk with the potential to improve personalized breast cancer risk assessment. Interestingly, the configuration of the weight map in our breast-anatomy-driven approach as indicated by the optimization experiments also suggests that the textural properties of different regions in the breast may contribute differently towards breast cancer risk, with dense tissue regions and the central breast quadrant having potentially a more important role. Further, the heterogeneity in textural measurements within the breast may also be important, as more than half of the features selected as strongest covariates in our model represent the variation (i.e., standard deviation) of the corresponding texture feature distribution within the breast.

In addition, our anatomy-driven approach to breast parenchymal texture analysis outperformed parenchymal texture features assessed *without* the incorporation of factors describing breast anatomy structure and variability^23^. We postulate that the improvement observed in this preliminary comparison is due to including factors that capture the wide variety of breast morphology and anatomy, found not only across the screening population but also within a single woman due to differences in positioning for FFDM. Incorporating such information, therefore, allows for also establishing anatomical correspondences across breasts of the same or different women, which in turn results in standardized imaging features and more comparable texture measurements. The observed improvement in discriminatory capacity may also be due to our polar grid, which allows for more granular texture measurements to be obtained in the retroareolar breast area where some of the most complex parenchymal tissue patterns usually appear^17^, and the ability to consider different contributions of the different sub-regions within the breast in the overall parenchymal texture signature. With this improvement, our anatomy-driven approach achieved a promising performance in this challenging task of breast cancer risk prediction with prior screening mammograms.

Overall, adding the breast-anatomy-driven features to baseline models with established breast cancer risk factors led to a significant increase in discriminatory capacity, suggesting a promising role in augmenting breast cancer risk assessment models. Similar conclusions have been reported in related studies^4,26^, where case-control classification models considering parenchymal textural features in addition to established risk factors and breast density achieved AUC values of 0.62 up to 0.78^10^. Together, these findings consistently support independent associations of parenchymal texture with breast cancer and, therefore, create a strong argument for incorporating quantitative breast textural features in models estimating breast cancer risk. The improvement of breast cancer risk estimation models can have substantial clinical implications, as it would allow for more informed recommendations for supplementary breast cancer screening (e.g., with magnetic resonance imaging or ultrasound) and prevention tailored by individual risk profiling. Hence, the potential of parenchymal texture to leverage breast cancer risk assessment might ultimately affect the chance of early cancer detection or prevention in women categorized as being at low breast cancer risk based on conventional risk factors

Important limitations of our study must also be noted. To avoid potential confounding effects of proprietary FFDM post-processing algorithms in textural measurements, our study focused only on “For Processing” (a.k.a., raw) FFDM images from a single vendor. In addition, our analysis was confined to a fixed feature set and, although elastic-net regression was used to alleviate model over-fitting, our reported model performance may be over-estimated due to the relatively small sample size as a single-institution evaluation. Considering the reported substantial differences in textural measurements across image acquisition settings^27^, different FFDM representations, and vendors^28^, in our future studies we will plan to more thoroughly test the robustness of our method by incorporating multiple FFDM vendors from larger populations. In addition, larger studies will allow us to more rigorously evaluate the added discriminatory capacity of such imaging biomarkers when considering additional demographic and clinical risk factors (*e.g*., age at menarche, parity, family history of breast cancer), potentially also expanding our feature set into higher phenotypic representations, including deep learning. Finally, while 2D FFDM images were analyzed as a first step for the purposes of this proof-of-concept study, we ultimately envision extending our algorithm to volumetric texture analysis for digital breast tomosynthesis images (also available for the studies in our study population), as tomosynthesis is increasingly being clinically implemented due to its reported improvements in sensitivity and specificity as compared to conventional 2D FFDM^29^. This new pseudo-3D imaging technology may, therefore, also result in superior imaging phenotypes of breast cancer risk.

In conclusion, our study provides evidence that incorporating breast anatomy strengthens the associations of mammographic parenchymal phenotypes with breast cancer risk and suggests that anatomy-driven measurements of parenchymal texture could complement current established risk factors and quantitative breast density measures. This additional information reflecting breast anatomy structure has the potential to further refine individualized risk assessment and, therefore, advance tailored screening and prevention strategies for breast cancer.

## Electronic supplementary material

Supplementary Information

## Data Availability

The data generated during the current study are available from the corresponding author on reasonable request.
