# Supplementary material for: Incorporating Breast Anatomy in Computational Phenotyping of Mammographic Parenchymal Patterns for Breast Cancer Risk Estimation
Source: Sci Rep. 2018 Nov 30;8:17489. doi: 10.1038/s41598-018-35929-9 (PMC6269457; doi:10.1038/s41598-018-35929-9)
Supplement: Supplementary file 1 — Supplementary Information [file 41598_2018_35929_MOESM1_ESM.pdf]

Incorporating Breast Anatomy in Computational Phenotyping of Mammographic Parenchymal Patterns for Breast Cancer Risk Estimation

Aimilia Gastounioti PhD, Meng-Kang Hsieh MS, Eric Cohen MS, Lauren Pantalone BS, Emily F. Conant MD, Despina Kontos PhD*

Department of Radiology, Perelman School of Medicine, University of Pennsylvania, Philadelphia, PA, 19104

*Corresponding author: University of Pennsylvania, Department of Radiology
3700 Hamilton Walk, Rm D702 Richards Bldg.
Philadelphia, PA 19104
Ph: 215-746-4064, fx: 215-573-1811
Email: Despina.Kontos@uphs.upenn.edu

Supplementary Notes

N1. Descriptions of texture descriptors

Gray-level histogram features are 13 well-established first-order statistics[1], which were calculated from the gray-level intensity histogram of the image using 128 histogram bins[2, 3].

The co-occurrence features reflect the spatial relationship between pixels and summarize the information encoded by the gray-level co-occurrence matrix (GLCM)[4] which corresponds to the relative frequency with which two neighboring pixels, one with gray level i and the other with gray level j, occur in the image. Such matrices are a function of the distance (d) and the orientation (ϑ) between the neighboring pixels. In this study, the $M \times M$ GLCM matrices were estimated using $M = 128$ gray levels to balance computational precision with efficiency, $d = 1.1$ mm[2, 3], and the co-occurrence features were computed by averaging over four polar-grid-driven orientations. Specifically, for each region defined by the polar grid, the four orientations corresponded to offsets, 0°, 45°, 90° and 135°, from the angle between the center of the region

and the x-axis, i.e., the axis starting from the breast nipple posteriorly and extending perpendicular to the pectoralis muscle.

Run-length features capture the coarseness of a texture in specified directions[5, 6]. A run is defined as a string of consecutive pixels with the same gray-level intensity along a specific linear orientation. Fine textures tend to contain more short runs with similar gray-level intensities, while coarse textures have more long runs with different gray-level intensities. Similarly to the GLCM, a $M \times N$ run-length matrix is defined, representing the number of runs with pixels of gray-level intensity equal to i in [1, M] and length of run equal to j in [1, N] along a specific orientation, where N is the size of the region. In correspondence with the co-occurrence features, the run-length statistics were estimated for $M = 128$ gray levels[2, 3], and averaged over the same four polar-grid-driven orientations.

The structural features include (a) the edge-enhancing index, which is based on edge enhancing diffusion and it describes the directionality of flow-like structures within the image[7], (b) the local binary pattern (LBP), which captures intensity variations between central and neighboring pixels[8] and (c) the fractal dimension (FD), which reflects the degree of complexity and was estimated using the box-counting method[9].

The edge-enhancing index of an image I is defined as:

$$\mathrm{E}_\sigma(\mathrm{I}) = \left(\frac{\lambda_1 - \lambda_2}{\lambda_1 + \lambda_2 + \eta}\right)^2$$

where $\lambda_1$and λ_2 ($\lambda_1 > \lambda_2$) are eigenvalues of the diffusion tension matrix of I and η is a normalizing factor which was set equal to 5 for this application[2, 3].

The LBP at pixel (x_c, y_c) is estimated as:

$$LBP(x_c, y_c) = \sum_{p=0}^{P-1} q\left(I_p - I_c\right)2^P,\ \left(x_p, y_p\right) = \left[x_c + Q\cos\left(\frac{2\pi p}{P}\right), y_c - Q\,sin\left(\frac{2\pi p}{P}\right)\right]$$

where I_c and I_p are the gray level values for pixels (x_c, y_c) and $\left(x_p, y_p\right)$, respectively, and q is a function which attributes values zero and one for negative and non-negative inputs, respectively. The neighborhood of each pixel is defined in terms of size and number of neighborhood pixels by

the parameters Q and P, respectively, which were set equal to $Q = 1$ and $P = 8$ following previous optimization experiments[2, 3].

The FD estimation relies on the concept of self-similarity. The fractal dimension of a bounded set *S* in Euclidean *n*-space is defined as:

$$FD = \lim_{r \to 0} \frac{\log(N_r)}{\log(\frac{1}{r})}$$

where N_r is the least number of distinct copies of *S* in the scale r. In case of an image, where fractals are not deterministic, the FD is approximated by the box-counting dimension as follows. We consider a three-dimensional spatial surface, with (x, y) denoting pixel position on the image plane and the third coordinate, *z*, denoting the pixel gray level. The image plane is partitioned into non-overlapping blocks of size $s \times s$, with s being an integer corresponding to the scale of the block. On each block, there is a column of boxes of size $s \times s \times s'$, where s' is the height of each box and it is determined by s, the size of the image and the total number of gray levels. If we assign numbers to the boxes of a block and the minimum and maximum gray levels fall into the mth and l^{th} boxes, respectively, then the boxes covering the block are counted in the number as $n_B = l - m + 1$. By considering the contributions of all blocks, N_r is counted as the sum of n_r over all blocks. Following this process for different values of r, FD can, then, be estimated from the least-squares linear fit of $\log(N_r)$ versus $\log(\frac{1}{r})$.

N2. Mathematical definition of the weight map

For a region with center *p*, we calculate the weight *S* based on the distance from the center of the region to the CBA (r_{CB}) and UOA (r_{UO}) centroids:

$$D_i(p) = a\frac{r_{CBmax} - r_{CB}(p)}{r_{CBmax}} + (1 - a)\frac{r_{UOmax} - r_{UO}(p)}{r_{UOmax}} \rightarrow S(p) = \frac{D_i(p)}{D_{imax}} \in [0,1] \quad (1)$$

where r_{CBmax} and r_{UOmax} are the maximum values of r_{CB} and r_{UO} over all regions, and a is a parameter which tunes the role of CBA versus UOA. For *T*, we utilize the density cluster (*DC*) map generated by LIBRA, with the binary parameter *b* indicating whether the weight map will weigh more heavily areas of dense or areas of fatty tissue:

$$T(p) = \begin{cases} \dfrac{DC(p)}{DC_{max}}, b = 1 \\ \dfrac{DC_{max} - DC(p)}{DC_{max}}, b = 0 \end{cases} \in [0,1] \quad (2)$$

where DC_{max} is the maximum value of DC over all regions. These two components are merged into the final weight map using equation (3), where parameter c tunes the relative importance of weights S and T:

$$M(p) = c * S(p) + (1 - c) * T(p) \rightarrow W(p) = \frac{M(p)}{M_{max}} \in [0,1] \quad (3)$$

Essentially, this weight map allows our methodology to code for the location, i.e. anatomical position, and the underlying tissue composition, i.e. dense versus fatty tissue, of each region of the breast. The how much each area is weighted in our final model was determined by the optimization process itself, as described in the next section.

N3. Optimization of breast-anatomy-driven feature parameterization

For all optimization steps, the following analysis was applied to the z-scored, averaged bilateral texture measurements. First, we identified pairs of features with absolute Pearson correlation greater than 0.90 and for each pair we removed the feature with the lowest variability in terms of its interquartile range (IQR)[10]. Starting from the remaining features, we built a logistic regression model using elastic net regression[11], a regularized technique suitable for correlated features which mixes penalization of the $L2$ and $L1$ norms and performs feature selection during model construction by assigning zero coefficients to weak covariates. To limit potential over-fitting, nested cross validation[12] was applied; first, the tuning hyper-parameters λ, that controls the penalty aggressiveness, and alpha, that controls the mixing of the $L2$ versus $L1$regularization, were chosen with ten-fold cross-validation (inner loop). The optimal combination of alpha and λ was selected as the one with deviance within one standard error of the model with minimum cross-validation deviance, and corresponded to the most parsimonious model[13]. With these optimal model hyper-parameters fixed, the performance of the model was then evaluated with leave-one-out cross validation (outer loop).

Using the statistical analysis described above, the first optimization step was to find an optimal value for D. We fixed the other four tunable parameters at f = 0.8, a = 0.5, b = 1, and c = 0.5, which is a combination of medium values balancing the roles of the anatomical components.

Using these fixed values we evaluated values D = 6.3 mm, 9.5 mm, 12.7 mm, 15.9 mm, 19.1 mm, 22.3 mm, and 25.5 mm, a range previously also investigated for the optimization of a square grid for parenchymal texture analysis[2, 14]. Fixing D at the value resulting in the highest AUC, we then performed a grid search optimization for all possible combinations of parameters f in [0.5, 1], a in [0, 1], b = 0 or 1, and c in [0, 1]. At each search round, the parameter combination being evaluated was the same across all images.

Case-control discriminatory performance increased with the polar grid's spatial density, giving an optimum value of D = 6.3 mm with AUC = 0.60, 95% CI [0.56, 0.63]. The grid search optimization for the remaining parameters using the optimal value of D = 6.3 mm showed that performance of the anatomy-driven framework varied substantially (with AUCs from 0.57 to 0.63) over different parameter combinations. The relation of discriminatory capacity to parameter values was consistent: stronger for $f = 0.8$, $0.7 \leq a \leq 0.8$, $b = 1$, and $c \leq 0.4$. Optimal performance (AUC=0.63, 95% CI [0.59 0.69]) was observed for $f = 0.9$, $a = 0.8$, $b = 1$, and $c = 0.4$, where elastic net regression (alpha = 1 and $\lambda = 0.16$) selected 30 textural features.

Supplementary Table

Supplemental Table S1. Associations with breast cancer risk and case-control discriminatory capacity. Odds ratios (ORs) per standard deviation increase in the standard risk factors and optimized breast-anatomy-driven texture features. Features shown reflect the ones selected by elastic net regression. Also shown: *p*-values, 95% confidence intervals (CIs), cross-validated discriminatory capacity (AUC).

	**OR**	***p*-value**	**95% CI**		**AUC**
Model based only on breast-anatomy-driven texture features					
TF1_mean	0.62	0.165	[0.32	1.22]	
TF6_mean	7.95	<0.001	[2.62	24.12]	
TF11_mean	0.39	0.052	[0.15	1.01]	
TF12_mean	1.32	0.412	[0.68	2.54]	
TF14_mean	0.41	0.112	[0.13	1.23]	
TF15_mean	1.57	0.374	[0.58	4.26]	
TF17_mean	0.56	0.090	[0.29	1.09]	
TF19_mean	1.09	0.853	[0.44	2.72]	
TF20_mean	1.62	0.090	[0.93	2.81]	
TF27_mean	0.92	0.788	[0.48	1.74]	
TF28_mean	1.05	0.927	[0.35	3.16]	
TF29_mean	0.42	0.128	[0.14	1.28]	
TF32_mean	1.09	0.637	[0.75	1.58]	
TF34_mean	0.93	0.796	[0.54	1.61]	
TF1_std	1.81	0.013	[1.13	2.89]	0.63
TF3_std	0.30	0.022	[0.11	0.84]	95% CI [0.59 0.69]
TF5_std	0.66	0.074	[0.43	1.04]	*p*-value = 0.041[a]
TF7_std	1.11	0.858	[0.36	3.46]	
TF11_std	0.26	0.003	[0.11	0.64]	
TF14_std	1.98	0.010	[1.18	3.32]	
TF15_std	0.92	0.652	[0.65	1.31]	
TF16_std	1.44	0.344	[0.68	3.04]	
TF20_std	1.97	0.015	[1.14	3.40]	
TF23_std	0.32	0.068	[0.10	1.09]	
TF26_std	1.85	0.055	[0.99	3.47]	
TF27_std	3.30	0.000	[1.73	6.32]	
TF28_std	1.57	0.196	[0.79	3.10]	
TF31_std	0.53	0.019	[0.31	0.90]	
TF33_std	0.45	<0.001	[0.29	0.70]	
TF34_std	1.01	0.960	[0.60	1.72]	
Model based on breast-anatomy-driven texture features, adjusted for standard risk factors					
Quantra VPD	1.03	0.378	[0.96	1.10]	
BMI	0.99	0.695	[0.94	1.04]	
Age	1.04	0.003	[1.01	1.06]	
TF1_mean	0.59	0.142	[0.29	1.19]	
TF6_mean	8.74	<0.001	[2.63	29.09]	0.67
TF11_mean	0.47	0.135	[0.17	1.27]	95% CI [0.60 0.72]
TF12_mean	1.35	0.379	[0.69	2.64]	
TF14_mean	0.46	0.186	[0.15	1.45]	
TF15_mean	2.18	0.151	[0.75	6.32]	
TF17_mean	0.57	0.099	[0.30	1.11]	

TF19_mean	1.03	0.951	[0.40	2.63]
TF20_mean	1.59	0.114	[0.90	2.82]
TF27_mean	0.86	0.645	[0.44	1.65]
TF28_mean	1.00	0.996	[0.33	3.05]
TF29_mean	0.47	0.180	[0.15	1.42]
TF32_mean	1.09	0.669	[0.73	1.62]
TF34_mean	0.98	0.933	[0.56	1.70]
TF1_std	1.90	0.009	[1.17	3.10]
TF3_std	0.33	0.036	[0.11	0.93]
TF5_std	0.70	0.133	[0.44	1.12]
TF7_std	0.93	0.902	[0.28	3.03]
TF11_std	0.24	0.003	[0.09	0.60]
TF14_std	1.91	0.016	[1.13	3.24]
TF15_std	0.92	0.663	[0.65	1.32]
TF16_std	1.47	0.321	[0.69	3.15]
TF20_std	2.04	0.012	[1.17	3.55]
TF23_std	0.33	0.086	[0.09	1.17]
TF26_std	1.85	0.056	[0.98	3.48]
TF27_std	3.32	<0.001	[1.71	6.45]
TF28_std	1.54	0.226	[0.77	3.09]
TF31_std	0.51	0.016	[0.30	0.88]
TF33_std	0.46	0.001	[0.29	0.72]
TF34_std	0.99	0.966	[0.58	1.69]

[a] For difference in AUC from the augmented model, including breast-anatomy-driven texture features and standard risk factors, by DeLong’s test.

Supplementary Figure

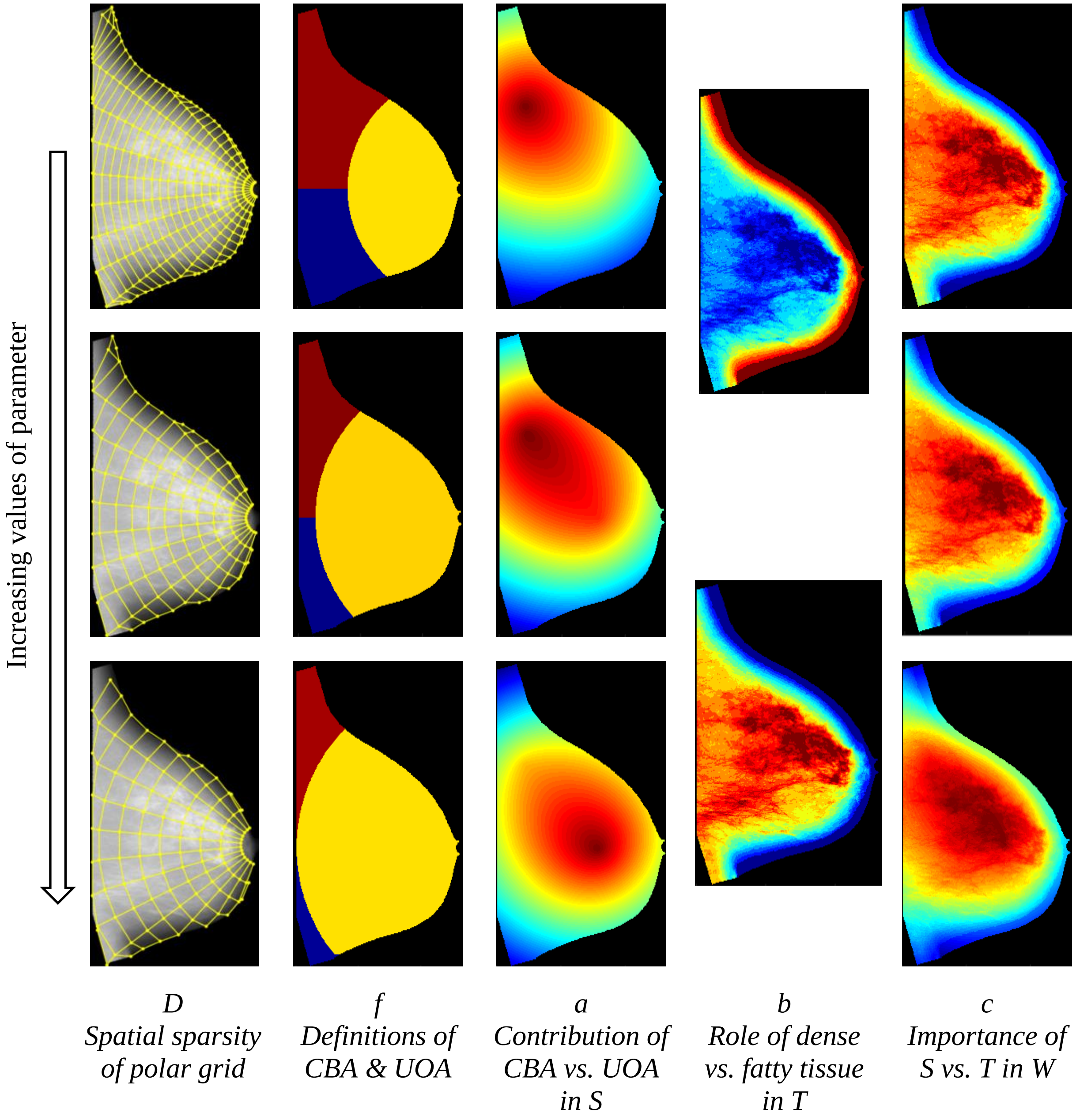

Supplemental Figure S1. Optimization parameters and their roles in breast-anatomy-driven texture analysis.

References

1. Materka A, Strzelecki M. Texture analysis methods–a review. Technical university of lodz, institute of electronics, COST B11 report, Brussels. 1998:9-11.
2. Keller BM, Oustimov A, Wang Y, Chen J, Acciavatti RJ, Zheng Y, et al. Parenchymal texture analysis in digital mammography: robust texture feature identification and equivalence across devices. Journal of Medical Imaging. 2015;2(2):024501-.
3. Gastounioti A, Oustimov A, Keller BM, Pantalone L, Hsieh M-K, Conant EF, et al. Breast parenchymal patterns in processed versus raw digital mammograms: A large population study toward assessing differences in quantitative measures across image representations. Medical Physics. 2016;43(11):5862-77.
4. Haralick RM, Shanmugam K, Dinstein IH. Textural features for image classification. Systems, Man and Cybernetics, IEEE Transactions on. 1973(6):610-21.
5. Galloway MM. Texture analysis using gray level run lengths. Computer graphics and image processing. 1975;4(2):172-9.
6. Chu A, Sehgal CM, Greenleaf JF. Use of gray value distribution of run lengths for texture analysis. Pattern Recognition Letters. 1990;11(6):415-9.
7. Weickert J. Coherence-enhancing diffusion filtering. International Journal of Computer Vision. 1999;31(2-3):111-27.
8. Ojala T, Pietikäinen M, Mäenpää T. Multiresolution gray-scale and rotation invariant texture classification with local binary patterns. Pattern Analysis and Machine Intelligence, IEEE Transactions on. 2002;24(7):971-87.
9. Caldwell CB, Stapleton SJ, Holdsworth DW, Jong RA, Weiser WJ, Cooke G, et al. Characterisation of mammographic parenchymal pattern by fractal dimension. Physics in Medicine & Biology. 1990;35(2):235-47.
10. Wang C, Brentnall AR, Cuzick J, Harkness EF, Evans DG, Astley S. A novel and fully automated mammographic texture analysis for risk prediction: results from two case-control studies. Breast Cancer Research. 2017;19(1):114. doi: 10.1186/s13058-017-0906-6.
11. Zou H, Hastie T. Regularization and variable selection via the elastic net. Journal of the Royal Statistical Society: Series B (Statistical Methodology). 2005;67(2):301-20.
12. Cawley GC, Talbot NL. On over-fitting in model selection and subsequent selection bias in performance evaluation. Journal of Machine Learning Research. 2010;11(Jul):2079-107.
13. Hastie T, Tibshirani R, Friedman JH. The Elements of Statistical Learning: Data Mining, Iference, and Prediction. 2nd ed: Springer; 2003.
14. Zheng Y, Keller BM, Ray S, Wang Y, Conant EF, Gee JC, et al. Parenchymal texture analysis in digital mammography: A fully automated pipeline for breast cancer risk assessment. Medical Physics. 2015;42(7):4149-60.
